# A U-shaped non-linear association between serum uric acid levels and the risk of Hashimoto’s thyroiditis: a cross-sectional study

**DOI:** 10.3389/fendo.2025.1514857

**Published:** 2025-02-04

**Authors:** Manli Yan, Wenhua Shi, Ping Gong, Yunsi Xie, Kaiyuan Zhang, Xiang Li, Hua Wei

**Affiliations:** ^1^ Second Clinical Medical College, Guangzhou University of Chinese Medicine, Guangzhou, China; ^2^ Department of Orthopedics, Guangdong Provincial Hospital of Chinese Medicine, Guangzhou, China; ^3^ Department of Endocrinology, Guangdong Provincial Hospital of Chinese Medicine, Guangzhou, China; ^4^ State Key Laboratory of Dampness Syndrome of Chinese Medicine, The Second Affiliated Hospital of Guangzhou University of Chinese Medicine, Guangzhou, China

**Keywords:** Hashimoto’s thyroiditis, non-diabetic adults, serum uric acid, RCS analysis, U-shaped curve

## Abstract

**Objective:**

Previous studies have found that the relationship between metabolic indicators and Hashimoto’s thyroiditis (HT) in non-diabetic adults remains unclear. This study aims to explore the association between metabolic indicators and HT, providing new theoretical insights for the clinical management of HT.

**Methods:**

Clinical data were collected from 2,015 non-diabetic adults at Guangdong Provincial Hospital of Chinese Medicine. The relationship between metabolic indicators and HT was analyzed using SPSS 26.0, R (version 4.2.1), and Zstats.

**Results:**

Among the 2,015 non-diabetic adult participants included in the study, 1,877 were in the non-HT group, while 138 were in the HT group. Significant differences were observed in metabolic indicators, including serum uric acid (SUA), serum creatinine (SCr), albumin (ALB) and high-density lipoprotein cholesterol (HDL-C), between the two groups, with statistical significance. A binary logistic regression model was established, revealing that SCr had a significant impact in both univariate and multivariate analyses. To further investigate the relationship between metabolic indicators and HT, we conducted a restricted cubic spline (RCS) analysis. The results demonstrated a clear non-linear relationship between SUA and HT, both before and after adjustment (All P < 0.01). Therefore, based on the inflection points derived from the RCS analysis, a segmented logistic regression analysis was performed. The findings indicated a significant association between both low and high levels of SUA and HT (Lower OR: 2.043; 95% CI: 1.405-3.019; P < 0.001; Higher OR: 2.369; 95% CI: 0.998-4.999; P = 0.034).

**Conclusion:**

This study is the first to reveal a U-shaped association between SUA levels and the risk of HT, suggesting that maintaining SUA levels within the range of 359.0-540.0 μmol/L may help reduce the risk of HT occurrence. This finding provides a new perspective for early intervention and long-term management of HT, particularly in terms of SUA regulation in HT patients, which holds potential clinical value.

## 1 Introduction

Hashimoto’s thyroiditis (HT), also known as chronic lymphocytic thyroiditis or autoimmune thyroiditis, is a chronic inflammatory disease of the thyroid with an etiology that remains incompletely understood. According to a meta-analysis (1), the global prevalence of HT among adults is 7.5%, with women being approximately four times more likely to be affected than men.

In HT, the immune system mistakenly targets the thyroid gland (2), producing antibodies such as anti-thyroid peroxidase antibody (TPOAb) and thyroid globulin antibody (TgAb), leading to persistent lymphocytic infiltration and chronic inflammation of the thyroid tissue, which results in specific thyroid dysfunction (3). As an autoimmune disease, the immune attack and antibody production are ongoing and often coexist with other autoimmune disorders (3), such as type 1 diabetes (3) and systemic lupus erythematosus (4).

Long-term immune activation may promote abnormal proliferation of lymphocytes, making HT a significant risk factor for primary thyroid lymphoma (5) and thyroid carcinoma (6), being associated with over 90% of thyroid lymphoma cases. Furthermore, persistent autoimmune responses can lead to severe central nervous system complications, such as Hashimoto’s encephalopathy (7). If not treated promptly, these complications may result in irreversible neurological damage (8). Therefore, early treatment of HT plays a crucial role in preventing complications and improving patient outcomes.

The late-stage glycation end products (AGEs) formed under chronic hyperglycemic conditions are evaluated as potential new biomarkers of oxidative stress (9). Elevated levels of oxidative stress-mediated by AGEs can induce damage to thyroid follicular cells, trigger thyroid inflammation and ultimately promote the development of HT (10). Research indicates that (11) the expression of IL-23 in thyroid follicular cells of HT patients is increased, contributing to autophagy suppression and the accumulation of reactive oxygen species (ROS). Additionally, elevated levels of TPOAb (12) in HT patients are also believed to be associated with increased concentrations of interleukin-6 and tumor necrosis factor-alpha, which play significant roles in the pathogenesis of insulin resistance (13). Therefore, some researchers suggest that TPOAb may exacerbate insulin resistance by promoting chronic inflammation, thereby interfering with normal glucose metabolism.

HT patients often experience dyslipidemia (14). Liu et al. (15)found a significant positive correlation between TPOAb levels and total cholesterol, triglycerides, insulin resistance and high-sensitivity C-reactive protein concentrations. Several studies (16–18) also suggest that TPOAb may serve as a potential link between HT and dyslipidemia. Previous research (19, 20) indicates that both hypothyroid and hyperthyroid patients may have an increased risk of developing hyperuricemia(HUA). The relationship between HT and metabolic disorders extends beyond the direct effects of hypothyroidism (21); autoimmune responses and chronic inflammation may play a more active role in the metabolic disturbances observed in HT patients (22). There is growing evidence that (23) thyroid hormones and the immune system interact in a complex bidirectional manner. As modulators of immune responses, thyroid hormones may ultimately lead to functional abnormalities through immune-mediated mechanisms.

Diabetic patients are often accompanied by metabolic disorders, including dyslipidemia (24). In addition, a strong correlation between diabetes and its related complications with elevated uric acid levels has been well established (25). To further investigate the relationship between HT and metabolic disturbances, we decided to select a non-diabetic population as the study group. Previous studies have shown that the relationship between metabolic-related indicators and non-diabetic populations with HT remains unclear. This study aims to explore the relationship between HT and metabolic products, providing a stronger theoretical basis for the long-term management of HT patients.

## 2 Materials and methods

### 2.1 Data collection

The data used in this study were collected from adult participants who underwent health examinations at Guangdong Provincial Hospital of Chinese Medicine from January 2023 to December 2023, with complete datasets. After evaluating the availability of laboratory data, including age, gender, glycated hemoglobin (HbA1c), fasting plasma glucose (FPG), lipid profiles (triglycerides (TG), total cholesterol (TC), high-density lipoprotein cholesterol (HDL-C), non-HDL cholesterol (nonHDL-C) and low-density lipoprotein cholesterol (LDL-C)), serum creatinine (SCr) and serum uric acid (SUA), participants meeting the screening criteria for non-diabetic adults were included (specifically, those aged over 18 years, with HbA1c levels below 6.0% and FPG below 6.1 mmol/L). A total of 2,015 participants were included in the study (1,164 males and 851 females).

According to the 2008 Guidelines for the Diagnosis and Treatment of Thyroid Diseases in China and the American Thyroid Association’s handbook on Hashimoto’s thyroiditis (26), participants with positive TPOAb and TgAb were defined as the Hashimoto’s thyroiditis group (HT group). Participants with negative serum TPOAb and TgAb were defined as the non-Hashimoto’s thyroiditis group (Non-HT group).

### 2.2 Statistical analysis

We employed univariate and multivariate logistic regression models to analyze the association between metabolic-related indicators and HT in a non-diabetic adult population. In evaluating the goodness of fit for the multivariable logistic regression model, we used the Hosmer-Lemeshow test. The results showed that the model fits well. To further assess the performance of the model, we introduced the concordance index as an evaluation metric. In this study, the concordance index of the constructed multivariable logistic regression model was 0.64 (95% confidence interval: 0.59-0.69). To further assess the impact of these indicators on the risk of HT, we constructed a multivariate logistic regression model based on restricted cubic splines (RCS). RCS is an effective strategy for analyzing the relationship between the risk of disease occurrence and independent variables, particularly suitable for exploring non-linear associations. Restricted cubic splines utilize smooth connections of polynomial functions to avoid assuming a linear relationship between covariates and the response variable. To avoid overfitting, the model was selected based on the minimum Akaike Information Criterion (AIC). The AIC value was smallest when the number of nodes was set to 4. Therefore, we used a restricted cubic spline function with 4 nodes at the 5th, 35th, 65th, and 95th percentiles to flexibly model the association between metabolic markers and HT. A P-value for non-linearity < 0.05 was defined as evidence of a non-linear relationship between the two. Additionally, the RCS model can identify risk inflection points (thresholds), defined as the values that minimize the odds ratio (OR). Once the thresholds were established, we conducted piecewise logistic regression analyses to explore the relationships between relevant indicators and HT across different intervals. The chi-squared test was used for categorical variables, while non-parametric tests were employed for continuous variables. All statistical tests were two-sided, with P < 0.05 considered statistically significant. All statistical analyses were performed using SPSS 26.0, R (version 4.2.1), and the “Zstats” package.

## 3 Results

### 3.1 Participant characteristics

A total of 2,015 non-diabetic adult participants were included. Among them, 138 participants were in the HT group, while 1,877 were in the Non-HT group. There were no significant differences in age and sex between the two groups. Notable differences with statistical significance were observed in metabolic-related indicators such as SUA, SCr, ALB and HDL-C between the two groups. In terms of thyroid-related indicators, the HT group showed significantly higher levels of TSH, TPOAb and TgAb compared to the Non-HT group, with these differences also being statistically significant (see **Table 1**).

**Table 1:** Comparison of baseline characteristics in the non-diabetic population.

	Total (n=2015)	Non-HT group (n=1877)	HT group (n=138)	*P-*value
Age (median (IQR))	45.0 (38.0, 52.0)	45.0 (38.0, 52.0)	44.0 (38.0, 52.0)	0.519
Sex (%)				0.929
Male	57.8%	57.8%	57.2%	
Female	42.2%	42.2%	42.8%	
HbA1c (median (IQR))	5.50 (5.20, 5.70)	5.50 (5.20, 5.70)	5.40 (5.20, 5.70)	0.314
FPG (median (IQR))	5.04 (4.78, 5.33)	5.03 (4.78, 5.32)	5.12 (4.83, 5.41)	0.087
SUA (median (IQR))	359.00 (297.00, 429.50)	361.00 (299.00, 432.00)	325.50 (271.75, 414.50)	0.001
SCr (median (IQR))	72.00 (59.00, 84.00)	72.00 (59.00, 84.00)	62.00 (55.00, 76.75)	<0.001
ALB (median (IQR))	46.40 (44.80, 48.10)	46.40 (44.80, 48.10)	46.00 (44.40, 47.58)	0.013
TG (median (IQR))	1.19 (0.87, 1.75)	1.20 (0.87, 1.76)	1.12 (0.84, 1.65)	0.121
TC (median (IQR))	5.15 (4.55, 5.76)	5.14 (4.55, 5.74)	5.19 (4.50, 5.90)	0.482
HDL-C (median (IQR))	1.37 (1.16, 1.62)	1.36 (1.15, 1.62)	1.44 (1.23, 1.69)	0.003
nonHDL-C (median (IQR))	3.74 (3.12, 4.38)	3.75 (3.13, 4.38)	3.70 (3.10, 4.34)	0.927
LDL-C (median (IQR))	3.22 (2.67, 3.77)	3.22 (2.67, 3.76)	3.25 (2.73, 3.84)	0.686
FT3 (median (IQR))	5.39 (5.00, 5.76)	5.41 (5.02, 5.77)	5.13 (4.77, 5.54)	<0.001
FT4 (median (IQR))	15.44 (14.25, 16.80)	15.46 (14.28, 16.82)	15.10 (14.01, 16.43)	0.052
TSH (median (IQR))	1.84 (1.33, 2.51)	1.82 (1.33, 2.48)	2.24 (1.51, 3.11)	<0.001
TPOAB (median (IQR))	28.00 (28.00, 29.17)	28.00 (28.00, 28.00)	1,095.30 (319.47, 1,300.00)	<0.001
TGAB (median (IQR))	1.30 (1.30, 1.79)	1.30 (1.30, 1.30)	85.18 (26.63, 206.37)	<0.001

### 3.2 Univariate and multivariate logistic regression analysis

We established a binary logistic regression model, incorporating variables with P < 0.05 into both univariate and multivariate logistic regression analyses (see **Table 2**). The influence of SCr was significant in both the univariate and multivariate analyses (univariate analysis: OR 0.976; 95% CI 0.965–0.987; multivariate analysis: OR 0.979; 95% CI 0.966–0.993; both P < 0.01). The impacts of SUA, ALB and HDL-C were not significant in the multivariate analysis (P > 0.05), indicating that their associations with the outcome diminished after controlling for other variables.

**Table 2:** Binary logistic regression analysis of the relationship between metabolic-related indicators and HT in the non-diabetic population.

	Univariate analysis		Multivariate analysis	
	OR (95%CL)	*P-*value	OR (95%CL)	*P-*value
SUA	0.997 (0.996~0.999)	0.010	1.000 (0.998~1.003)	0.777
SCr	0.976 (0.965~0.987)	<0.001	0.979 (0.966~0.993)	0.004
ALB	0.916 (0.856~0.980)	0.011	0.944 (0.880~1.013)	0.108
HDL-C	1.799 (1.138~2.843)	0.012	1.359 (0.809~2.283)	0.246

### 3.3 Restrictive cubic spline identification of uric acid ranges related to minimum risk

**Figure 1:**
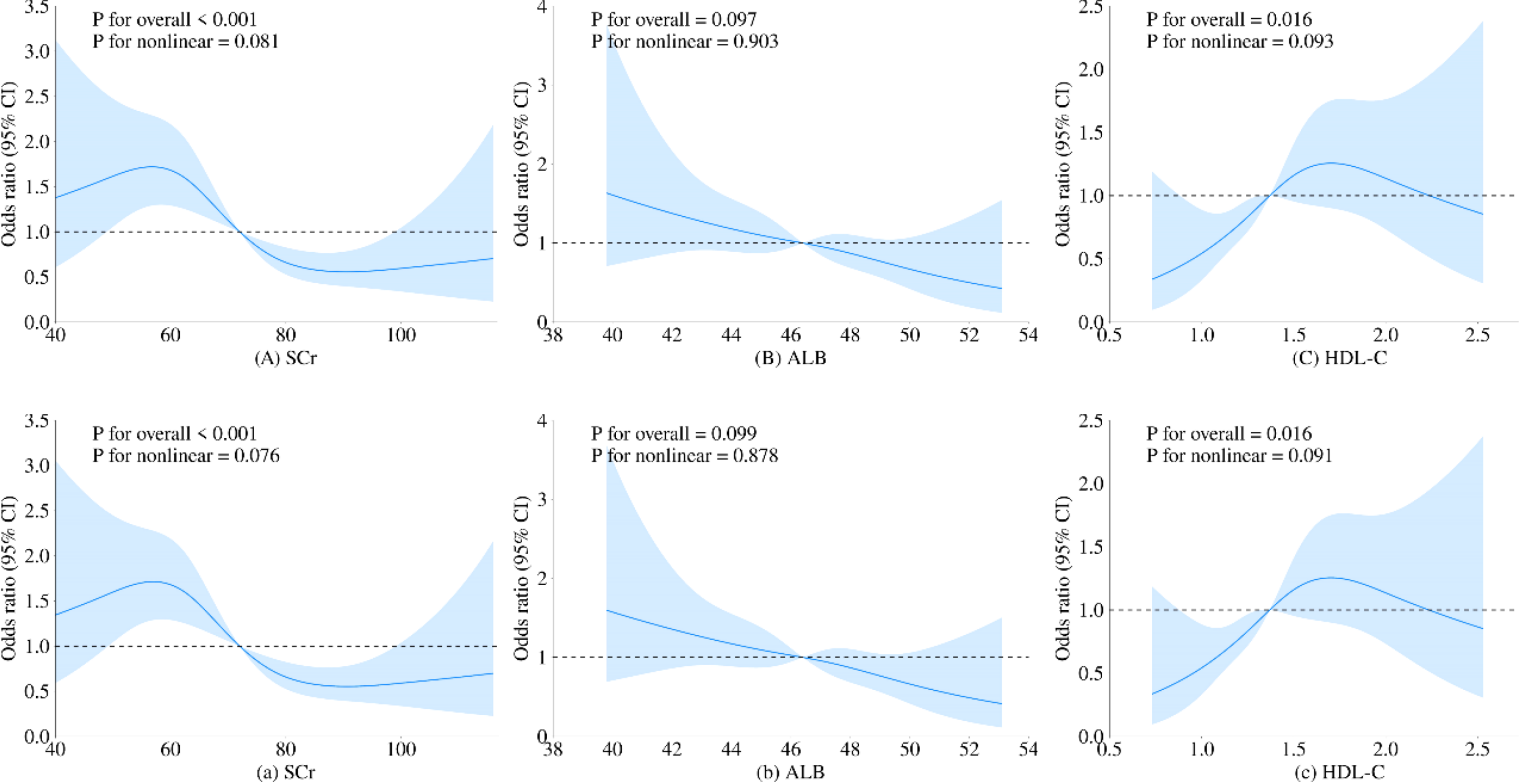
Restricted cubic spline regression analysis of the relationship between SCr, ALB, and HDL-C as continuous variables and HT. The blue solid line represents the estimated adjusted OR, while the shaded area indicates the 95% confidence interval. The dashed line represents an odds ratio or risk ratio of 1.0. Panels **(A–C)** show the spline curves for univariate regression, while subpanels (a-c) present the spline curves adjusted for age and gender.

**Figure 2:**
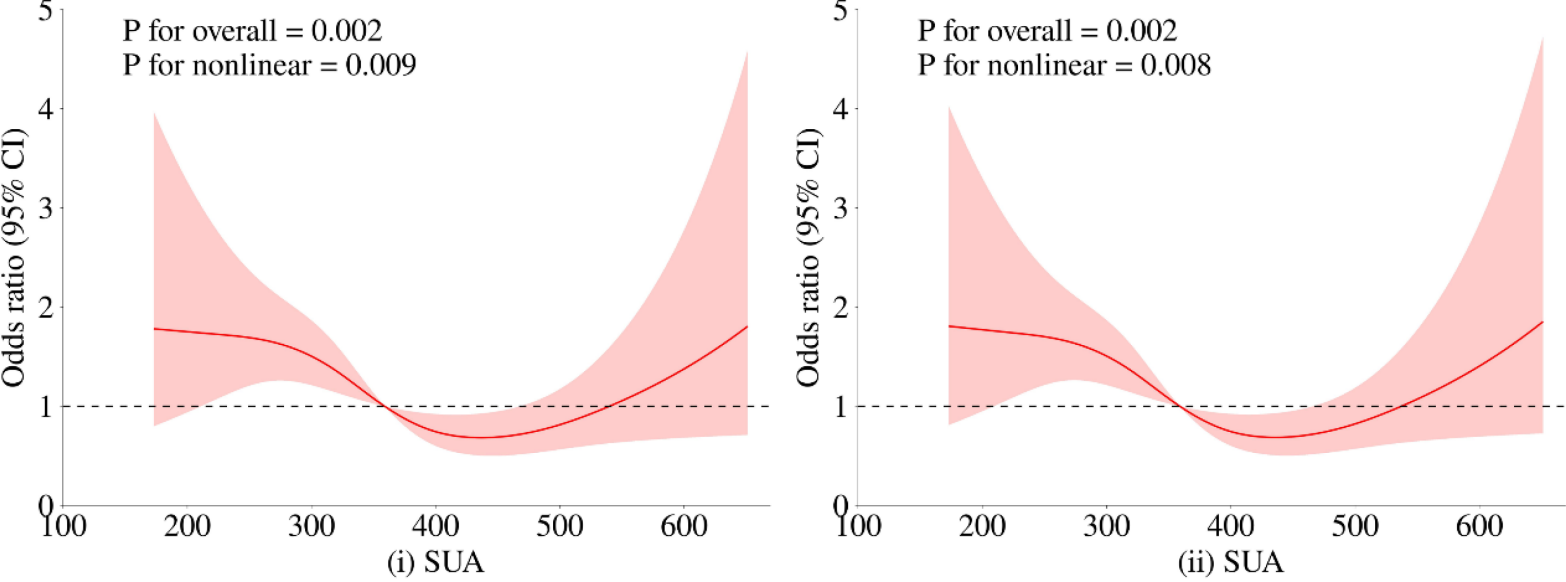
Restricted cubic spline regression analysis of the relationship between SUA as a continuous variable and HT. The red solid line represents the estimated adjusted OR, while the shaded area indicates the 95% confidence interval. The dashed line represents an odds ratio or risk ratio of 1.0. Panel (i) shows the spline curve for univariate regression, while panel (ii) presents the spline curve adjusted for age and gender.

To further investigate the relationship between metabolic-related indicators and HT, we conducted a RCS analysis (see **Figures 1**, **2**). The results showed that neither SCr, ALB, nor HDL-C demonstrated a significant non-linear relationship with HT in both univariate analyses and after adjusting for age and gender (all P > 0.05). In contrast, SUA exhibited a significant non-linear relationship with HT before and after adjustment (all P < 0.01). Therefore, based on the inflection points derived from the RCS, we further analyzed the “U” shaped relationship between SUA and HT. The reference range was set at 359.0-540.0 μmol/L, where the lower subgroup consisted of participants with SUA levels below 359.0 μmol/L, and the higher subgroup comprised those with SUA levels above 540.0 μmol/L. By establishing a segmented logistic regression model, we analyzed the relationship between SUA levels in different ranges and HT. Compared to the reference range, the adjusted logistic model indicated that both lower and higher SUA levels were significantly associated with HT (Lower OR: 2.043; 95% CI: 1.405-3.019; P < 0.001; Higher OR: 2.369; 95% CI: 0.998-4.999; P = 0.034) (see **Table 3**).

**Table 3:** Threshold effect analysis of SUA and HT in the non-diabetic population.

Outcome	Adjusted OR (95% CI)	*P*-value
Model 1 Fitting by standard linear model	0.997 (0.996-0.999)	0.01
Model 2 Fitting by three-piecewise linear model		
Reference interval (359.0-540.0 μmol/L)	[Reference]	
Lower (< 359.0 μmol/L)	2.043 (1.405-3.019)	<0.001
Higher (> 540.0 μmol/L)	2.369 (0.998-4.999)	0.034
*P* for log-likelihood ratio	0.003	

Adjusted for age and sex.

## 4 Discussion

To our knowledge, this is the first cross-sectional study investigating the relationship between metabolic-related indicators and HT in a non-diabetic population. Our findings reveal a significant non-linear U-shaped relationship between SUA and HT in this cohort.

UA (27) is primarily synthesized in the liver, intestines and vascular endothelium. It is produced endogenously through purine metabolism, catalyzed by enzymes, from damaged, dying and dead cells. Additionally, UA levels are influenced by the purine content in dietary intake (28).

The human body maintains homeostasis of UA concentration through a dynamic balance of production and excretion (29). However, when this balance is disrupted, it often results in elevated UA levels in the blood, leading to HUA.

UA is known to promote the elimination of reactive oxygen species (ROS), which contributes to its antioxidant properties (30). However, recent studies (31) have shown that the formation of UA is also accompanied by the generation of ROS. UA can stimulate the activity of NADPH oxidase while inhibiting the activity of endothelial nitric oxide synthase, consequently reducing the metabolism of nitric oxide (NO). Furthermore, UA enhances the affinity of arginase for L-arginine, which further promotes ROS production. Although ROS (32) plays a crucial role in regulating cellular signaling and physiological homeostasis, excessive production of ROS (32, 33) can lead to pathological states of oxidative stress. This overproduction has been shown to irreversibly alter cellular structure and function. Overall, the generation of ROS is vital for regulating appropriate immune responses.

The human gut (34) is the only site that continuously activates the immune system through direct contact with the microbiome. The production of ROS in the gut is considered a double-edged sword (34): it is an indispensable mechanism for defending against pathogens and facilitating mucosal healing, but excessive ROS production can adversely affect mucosal integrity and epithelial barrier function, and is even closely associated with the occurrence and progression of multisystem diseases.

HT is characterized by the infiltration of inflammatory cells into the thyroid gland and the production of antibodies against thyroid-specific antigens, which triggers a persistent state of immune inflammation (26). Chronic inflammation leads to progressive destruction and fibrosis of follicular cells, ultimately resulting in hypothyroidism. Recent studies suggest (35) that oxidative stress is a primary molecular driver of tissue damage and has garnered significant attention in the pathogenesis of autoimmune and inflammatory diseases. Under the influence of environmental and genetic factors, lymphocytes (36) participate in the pathogenesis of autoimmune diseases by producing autoantibodies and ROS.

Research by Virili et al. (37) has indicated that in patients with HT, transmission electron microscopy reveals changes in microvillus thickness and increased spacing between adjacent microvilli, along with ultrastructural morphological changes in the epithelial cells of the distal duodenum. Several studies (37, 38) have also found a close relationship between gut microbiota and thyroid function, as well as the risk of HT.

Approximately 30-40% (39) of SUA is excreted via the gut. Recent studies have suggested that gut clearance defects (40) are one of the significant causes of HUA, highlighting the important role of gut microbiota (41) in regulating UA metabolism. Research by Li, Tianhe et al. (42) has confirmed the feasibility of alleviating endocrine diseases by improving gut microbiota to reduce gut oxidative stress. UA is closely related to nutrition, immune inflammation, oxidative stress and gut microbiota, and these critical biological characteristics position UA as a key player in the pathogenesis of various diseases (43, 44).

In our study, we observed that SUA levels within a specific range (359-540 μmol/L) were significantly associated with a lower risk of HT. Therefore, maintaining SUA levels within this reference range may help reduce the incidence of HT, which is potentially linked to mechanisms involving oxidative stress and gut microbiota. This finding could provide important clinical recommendations for the prevention and management of HT.

## 5 Limitations

This study is a large-sample, single-center cross-sectional investigation; however, it does have some limitations. For instance, the sample size is relatively small, and the study population is drawn from a specific region and ethnic group, which may limit the generalizability of the findings. Additionally, the study did not consider individual-specific disease-related information, which could introduce bias into the results. Another limitation is that thyroid function was not categorized or analyzed in detail, as we considered immune dysregulation to be the primary driver of metabolic abnormalities. However, thyroid dysfunction could potentially amplify the effects of immune factors, or abnormal thyroid function itself may serve as an indicator of immune factor severity. This is an important aspect worth further discussion, which could lead to a more comprehensive understanding of the relationship between thyroid function, immune factors and metabolic disturbances.

## 6 Conclusion

This study is the first to explore the relationship between metabolic-related indicators and HT in a non-diabetic population. We found a significant non-linear U-shaped relationship between UA levels and the risk of HT. Specifically, lower risk of HT was significantly associated with UA levels within the range of 359-540 μmol/L. Our results suggest that maintaining UA levels within the reference range may help reduce the incidence of HT, potentially linked to mechanisms involving oxidative stress and gut microbiota regulation.

Despite limitations such as the relatively small sample size and the specificity of the regional and ethnic population, these findings provide important clinical insights for the prevention and management of HT. They emphasize the potential role of SUA in endocrine diseases, warranting further research and validation.

## Data Availability

The original contributions presented in the study are included in the article/supplementary material. Further inquiries can be directed to the corresponding authors.
